# Metabolomic Biomarkers in Blood Samples Identify Cancers in a Mixed Population of Patients with Nonspecific Symptoms

**DOI:** 10.1158/1078-0432.CCR-21-2855

**Published:** 2022-01-04

**Authors:** James R. Larkin, Susan Anthony, Vanessa A. Johanssen, Tianrong Yeo, Megan Sealey, Abi G. Yates, Claire Friedemann Smith, Timothy D.W. Claridge, Brian D. Nicholson, Julie-Ann Moreland, Fergus Gleeson, Nicola R. Sibson, Daniel C. Anthony, Fay Probert

**Affiliations:** 1 Medical Research Council Oxford Institute for Radiation Oncology, Department of Oncology, University of Oxford, Oxford, United Kingdom.; 2 Department of Radiology, Oxford University Hospitals NHS Foundation Trust, Oxford, United Kingdom.; 3 Department of Pharmacology, Medical Sciences Division, University of Oxford, Oxford, United Kingdom.; 4 Department of Neurology, National Neuroscience Institute, Singapore.; 5 Duke-NUS Medical School, Singapore.; 6 Nuffield Department of Primary Care Health Sciences, University of Oxford, Oxford, United Kingdom.; 7 Department of Chemistry, University of Oxford, Oxford, United Kingdom.

## Abstract

**Purpose:**

Early diagnosis of cancer is critical for improving patient outcomes, but cancers may be hard to diagnose if patients present with nonspecific signs and symptoms. We have previously shown that nuclear magnetic resonance (NMR) metabolomics analysis can detect cancer in animal models and distinguish between differing metastatic disease burdens. Here, we hypothesized that biomarkers within the blood metabolome could identify cancers within a mixed population of patients referred from primary care with nonspecific symptoms, the so-called “low-risk, but not no-risk” patient group, as well as distinguishing between those with and without metastatic disease.

**Experimental Design:**

Patients (*n* = 304 comprising modeling, *n* = 192, and test, *n* = 92) were recruited from 2017 to 2018 from the Oxfordshire Suspected CANcer (SCAN) pathway, a multidisciplinary diagnostic center (MDC) referral pathway for patients with nonspecific signs and symptoms. Blood was collected and analyzed by NMR metabolomics. Orthogonal partial least squares discriminatory analysis (OPLS-DA) models separated patients, based upon diagnoses received from the MDC assessment, within 62 days of initial appointment.

**Results:**

Area under the ROC curve for identifying patients with solid tumors in the independent test set was 0.83 [95% confidence interval (CI): 0.72–0.95]. Maximum sensitivity and specificity were 94% (95% CI: 73–99) and 82% (95% CI: 75–87), respectively. We could also identify patients with metastatic disease in the cohort of patients with cancer with sensitivity and specificity of 94% (95% CI: 72–99) and 88% (95% CI: 53–98), respectively.

**Conclusions:**

For a mixed group of patients referred from primary care with nonspecific signs and symptoms, NMR-based metabolomics can assist their diagnosis, and may differentiate both those with malignancies and those with and without metastatic disease.

*See related commentary by Van Tine and Lyssiotis, p. 1477*

**Translational Relevance**

The accurate and timely diagnosis of cancer is critical for reducing the morbidity and mortality associated with late-stage cancer diagnoses, but early diagnosis can be difficult to achieve if patients present with nonspecific symptoms that may be due to cancer, the so-called “low-risk, but not no-risk” patient group. This often presents a challenge to physicians, and tests that can help aid diagnosis are urgently needed. Here we have shown that biofluid metabolomics analysis of peripheral blood from a cohort of such patients can detect cancers with high sensitivity and specificity. Biofluid metabolomics is a cheap and easy-to-administer test, requiring only a small blood sample, and has the potential to act as an effective triage tool to prioritize patients with nonspecific symptoms for more invasive investigations or to rule out cancer in patients with nonspecific symptoms.

## Introduction

There is a strong correlation between earlier cancer diagnoses and improved outcomes (1). If cancers are detected early, they are more easily treated and have better outcomes as they are likely to be at an early stage, with less nodal involvement, and are less likely to have metastasized (2, 3).

Current cancer referral pathways, such as the “2-week wait” pathways in the UK, are designed predominantly around organ-specific symptoms, such as hemoptysis or hematuria, or clinically palpable abnormalities, such as breast lumps or an abdominal mass. This process is ideal for cancers that present with specific symptoms but is problematic when patients present with nonspecific symptoms, such as fatigue. If there are no organ-specific symptoms or signs it may be difficult to know to which specialist the patient should be referred. Time and resources may be wasted, leading to delays in diagnoses, in turn leading to increases in the proportion of patients presenting with advanced tumors (4).

To help identify cancers in patients with nonspecific symptoms, the Suspected CANcer (SCAN) pathway was established in Oxfordshire, UK, as part of the Accelerate, Coordinate, Evaluate (ACE) Wave 2 initiative, which developed multidisciplinary diagnostic center (MDC) based pathways for these patients (5). SCAN is a referral pathway from primary care to the hospital for patients with nonspecific symptoms such as fatigue and weight loss. All patients referred to SCAN undergo a contrast-enhanced CT of the chest, abdomen, and pelvis, blood biochemistry and hematology analysis. If an obvious pathology is not detected on initial investigations, consultant physician review of the patient at an MDC is used in an attempt to reach a diagnosis.

Biofluid metabolomics is potentially an alternative for this patient group. Biofluid metabolomics is a conceptually simple and inexpensive technique that relies upon the simultaneous determination of the levels of small-molecule constituents within a biological sample, which are analyzed to establish disease-specific patterns. These metabolomic profiles are produced by an analytical technique such as nuclear magnetic resonance (NMR) spectroscopy or mass spectrometry. We have previously demonstrated that we can use NMR-based biofluid metabolomic analysis to sensitively and specifically detect the presence of brain metastases in a mouse model of breast cancer (6). Moreover, our data indicate that there is a distinct pattern of metabolites that allows discrimination between brain and systemic tumor burdens (6). Other groups have further been able to identify different types of cancers using NMR metabolomics, including lung (7), colorectal (8), pancreatic (9), liver (10, 11), breast (12), and bladder (13) cancers.

Here, we hypothesized that biofluid metabolomics would (i) be able to distinguish patients with cancer from those without cancer, in a mixed cohort with nonspecific symptoms, and (ii) be able to distinguish patients with and without metastases. We have tested these hypotheses using NMR analysis of blood samples from patients recruited to the SCAN pathway.

## Materials and Methods

### Patient recruitment

Patients (*n* = 304) were recruited from the Oxfordshire (UK) SCAN pathway (5) between May 2017 and August 2018. Patients were recruited to the SCAN pathway from the general Oxfordshire population if they met three essential referral criteria: (i) there was no other “2-week wait” cancer-specific referral pathway suitable for the patient; (ii) the patient was ≥40 years old; (iii) the patient had at least one of the following: unexplained weight loss, severe unexplained fatigue, persistent nausea or appetite loss, new atypical pain, an unexplained laboratory test finding that did not suggest a specific diagnosis, a primary care physician clinical suspicion of cancer or serious disease (“gut feeling”; ref. 5). All patients gave written informed consent prior to study admission. The study was conducted in accordance with the UK policy framework for health and social care research and was approved by the Oxford Radcliffe Biobank (ORB) research tissue bank ethics committee (reference 19/SC/0173). Patients were included in the study if (i) both a blood sample and CT scan were available, (ii) a confirmed cancer/noncancer diagnosis was reached during the SCAN follow-up, and (iii) no contaminant peaks were observed in the NMR spectrum.

### Patient pipeline and biofluid sample collection

Patients were referred from primary care to the SCAN pathway without diagnoses known, ensuring random sampling from the population. Referred patients underwent a contrast-enhanced thorax, abdomen, and pelvis CT, a full blood count and blood-based biochemical analyses, after a minimum fasting period of 2 hours. If a significant pathology was not detected by initial investigations, each patient then underwent medical consultation in a MDC. The MDC recommended further investigations if indicated, to reach a definitive diagnosis (5). If a patient received a cancer diagnosis within 62 days (2 months) of initial assessment they were considered to have had their cancer at the time of initial investigation (14). Patients were followed up for 12 months in total after SCAN assessment, both to record the diagnosis from the SCAN pathway and to determine whether any cancers developed that were not diagnosed within the first 62 days.

Blood for metabolomics was collected into lithium-heparin tubes immediately prior to CT imaging and was left to stand at room temperature before separation by centrifugation (2,200 × *g*, 10 minutes). Plasma was immediately separated, aliquoted, and stored at −80°C.

### NMR acquisition

NMR metabolomics analysis of plasma was carried out as previously described (15, 16). Plasma samples from patients (*n* = 299, 150 μL each) were defrosted on ice and mixed with NMR buffer (450 μL, 70 mmol/L sodium phosphate at pH7.4 in D_2_O). Samples were clarified by centrifugation (16,000 × *g*, 3 minutes) to remove particulate matter before transferring to a 5-mm NMR tube.

All NMR spectra were acquired using a 700MHz Bruker AVIII spectrometer operating at 16.4T equipped with a ^1^H (^13^C/^15^N) TCI cryoprobe. Sample temperature was stable at 310K. ^1^H NMR spectra were acquired using both a 1D NOESY presaturation scheme with a 2-second presaturation for attenuation of the water resonance, as well as a Carr-Purcell-Meiboom-Gill (CPMG) spin-echo sequence to suppress broad signals arising from large molecular weight blood components (40-millisecond total effective filter time, 32 data collections, acquisition time of 1.5 seconds, relaxation delay of 2 seconds, fixed receiver gain; ref. 15). 2D ^1^H-^1^H total correlation spectroscopy (TOCSY) spectra were acquired on at least one sample in each classification to assign the metabolites. Assignments were confirmed by reference to literature values (17, 18) and online databases (19, 20), with spiking experiments. For quality control, pooled samples were spread throughout the run to monitor technical variation. Approximately 70 soluble metabolites, including a range of lipoprotein species, amino acids, carbohydrates, and ketone bodies, were detected, as previously described (21).

### NMR data preprocessing

All spectra were phased, baseline corrected, and referenced to lactate at δ = 1.33 ppm, followed by visual inspection for errors, spectral distortion, or contamination. Only CPMG spectra were used for statistical analyses. The region 0.20 to 9.68 ppm, excluding the region covering the residual water peak, was divided into 0.01-ppm width ‘buckets’, integrated, and Pareto scaled (more details in Supplementary Methods). Mean NMR spectra for groups of patients were prepared by summing individual processed spectra from a patient group, then dividing the summed spectrum by the number of patients. Difference spectrum is the mean solid tumor spectrum minus the mean noncancer spectrum.

### Statistical analysis

Prior to metabolomics analyses, patients were randomized into a modeling set (two thirds of patients, *n* = 192) and an independent test set (one third of patients, *n* = 92), based on referral order. The independent test set was reserved for determining the ability of the models to classify new patients.

NMR bucket integrals were imported into R (R Foundation for Statistical Computing, Vienna, Austria; RRID:SCR_001905; ref. 22). All multivariate statistical analysis was conducted using in-house R scripts and the *ropls* package (RRID:SCR_016888; ref. 23). Orthogonal partial least squares discriminatory analysis (OPLS-DA) was used to generate diagnostic mathematical models to classify patients in the modeling set employing the nonlinear iterative partial least squares (NIPALS) algorithm. The number of orthogonal components was computed using a seven-fold internal cross-validation to identify the number of components to produce the optimal Q^2^ up to a maximum of nine. The independent test set was not used at this stage. Within the modeling set, the quality of classification was assessed using a 10-fold external cross-validation scheme with 1,000 repetitions in total (to correct for unequal class sizes). This validation scheme involves multiple iterations of splitting the data into training and external-validation sets. The training data are used to estimate the model parameters and learn the underlying discriminatory patterns between the groups under consideration, whereas the external-validation sets are employed to assess the accuracy and generalizability of the trained models. We quantified the response of the ensemble of models by calculating the accuracy, sensitivity, and specificity of each model from the predicted classifications of the external-validation sets (i.e., samples that were not used in model building). It is important to appreciate that the classifier (OPLS-DA) was blinded to the external-validation set during the process of model training. These values were compared with those of a null distribution (obtained from randomly permuting the classifications) using the two-sided Kolmogorov–Smirnov test (significant if *P* value < 0.05). Discriminators were identified by calculating the variable importance of projection (VIP) score. Metabolites with a VIP > 2 were considered significant in the multivariate modeling. This validation scheme tends to avoid overfitting and helps assess the generalizability of the model to previously unseen datasets. For an exhaustive discussion on validation see ref. 24. The generalizability of these models was further confirmed using the independent test set which was left out of both model training and validation.

Following this exhaustive validation, receiver operator characteristic (ROC) curves were constructed to determine the optimal diagnostic threshold within the OPLS-DA model. The point closest to the top left corner of the ROC curve was used to determine the optimal overall sensitivity, specificity, and balanced accuracy [ (*sensitivity + specificity*)/2]. F_1_ values were calculated from ${F_1} = {\frac{{2{\mathrm{TP}}}}{{2{\mathrm{TP}} + {\mathrm{FP}} + {\mathrm{FN}}}}$ to provide an overall assessment of accuracy suitable for models with uneven group sizes.

This classification threshold was then used with the independent test set (i.e., the one third of patients reserved at the beginning of the study, unused until now). Classifications were made for each patient in the independent test set and 2 × 2 contingency tables constructed to determine efficacy of the models for predicting the identity of unknown samples. Fisher exact test was used to determine if this classification was significantly better than chance. Confidence intervals(CI) for classification metrics were calculated using the Wilson score interval (details in Supplementary Methods). Finally, patients who received a changed diagnosis within the first year were assessed to see if the model predicted them as having cancer.

Comparisons of ROC curves and male:female sex ratios were carried out by calculation of the *Z*-statistic for each pair of curves or ratio and comparing with the expected normal distributions (details in Supplementary Methods). After multivariate modeling, univariate statistical analyses were conducted by summing integrals for each metabolite resonance identified as significant in the multivariate models. Differences between means were calculated using the Student *t* test. Correlation analyses were carried out using Pearson's correlation.

### Data availability statement

The data generated in this study are available upon request from the corresponding author.

## Results

### Patient demographics and NMR acquisitions

**Figure 1.**
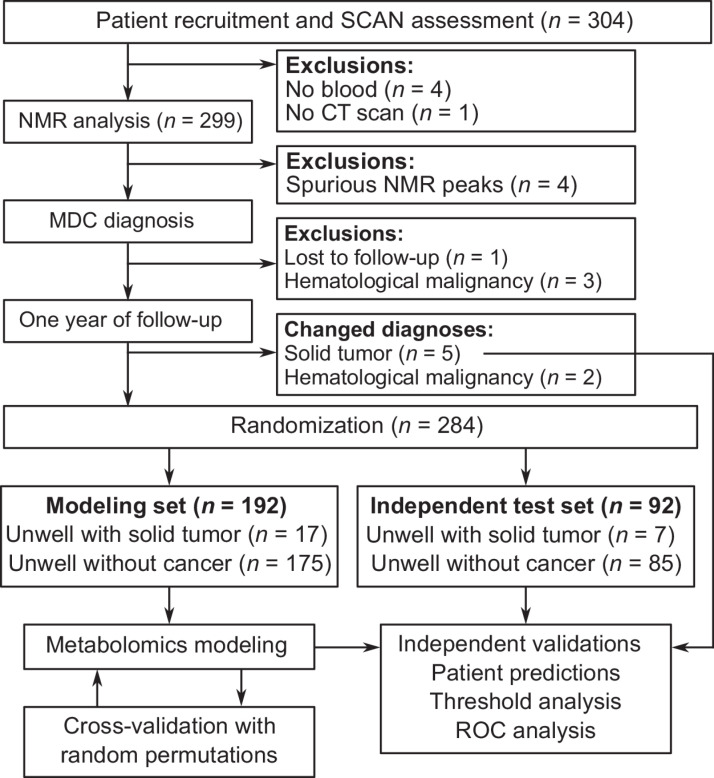
Study schematic showing patient recruitment into the study, exclusions, biofluid collection, and confirmed diagnoses.

In total, 304 patients were recruited from the SCAN pathway during the study period. Eleven patients (3.6%) were excluded from analyses owing to missing biofluid samples, lack of CT scan, loss to follow-up, NMR spectrum contamination, or lack of confirmed cancer/noncancer diagnosis. A flow chart of patient recruitment is given in Fig. 1.

The total numbers of patients with cancer diagnosed by the SCAN pathway during the study period was 27 of 294 (9.2%). SCAN was more successful at detecting new cancer diagnoses than existing organ-specific urgent cancer referral pathways (6.6% in 2019–20; ref. 25). Solid tumors were diagnosed in 24 of the 294 patients (8.2%). Hematologic malignancies were diagnosed in the remaining 3 patients, but these were removed from modeling owing to both the low number and alternate diagnosis strategies available for these diseases. The most common solid tumors diagnosed were large bowel (*n* = 8) and lung (*n* = 5). The remaining cancers were found in numerous organs and are listed in Supplementary Table S1. Within the population that received solid tumor diagnoses, 8 patients (33%) had nonmetastatic cancers, and 16 patients (66%) had metastatic cancer. Seven patients who received a noncancer diagnosis from the MDC assessment received a cancer diagnosis within 1 year. Of these, 5 received solid tumor diagnoses and 2 received hematologic malignancy diagnoses.

The mean patient age was 68 years. Patients receiving solid tumor diagnoses were older (73 ± 10 years) than unwell patients that received a noncancer diagnosis (68 ± 12 years, *P* < 0.05). No other subgroup analysis showed significant age differences. More women than men were recruited (*P* < 0.001), but no difference was evident in the distribution of men and women between the model-building and test sets. The mean patient body mass index (BMI) was 26.0 ± 6.2 kg/m^2^; and no differences in BMI were found between different patient groups. Full patient demographic details are summarized in Table 1 and a visual representation of BMI across the patient population is included in Supplementary Fig. S1. The most common reasons for patient referral were weight loss (64%), general practitioner gut feeling (63%), unexplained laboratory results (37%), fatigue (29%), nonspecific pain (28%), and nausea/appetite loss (27%). On average, patients presented with symptoms in 2 ± 1 of these six categories.

**Table 1.** Patient demographics.

Variables	Total	Solid tumor diagnoses	Noncancer diagnoses	Modeling set	Independent test set
Number of patients	284 (100%)	24 (8%)	260 (92%)	192 (68%)	92 (32%)
Age					
Mean ± SD	68 ± 12	73 ± 10^a^	68 ± 12	69 ± 11	67 ± 13
(Minimum, maximum)	(40, 93)	(53, 93)	(40, 90)	(40, 90)	(44, 93)
BMI (kg/m^2^)					
Mean ± SD	26.0 ± 6.2	28.1 ± 4.6	25.8 ± 6.3	25.8 ± 6.3	26.3 ± 6.0
(Minimum, maximum)	(13.2, 57.1)	(18.3, 38.4)	(13.2, 57.1)	(13.2, 57.1)	(15.4, 45.7)
Sex					
Male	123 (43%)	10 (42%)	113 (43%)	78 (41%)	45 (49%)
Female	161 (57%)^b^	14 (58%)	147 (57%)^b^	114 (59%)^b^	47 (51%)

Note: Data are presented as *n* (%), or as mean ± SD with range (minimum, maximum) for age and BMI (BMI data were available for 278 out of the 284 patients). Differences between groups were compared using Student *t* test for age and calculation of Z-statistic for sex.

^a^ *P* < 0.05 compared with noncancer diagnoses.

^b^ *P* < 0.001, male:female ratio differs from 1:1.

### Plasma metabolomics identifies cancer in individuals with nonspecific symptoms with high sensitivity and specificity

**Figure 2.**
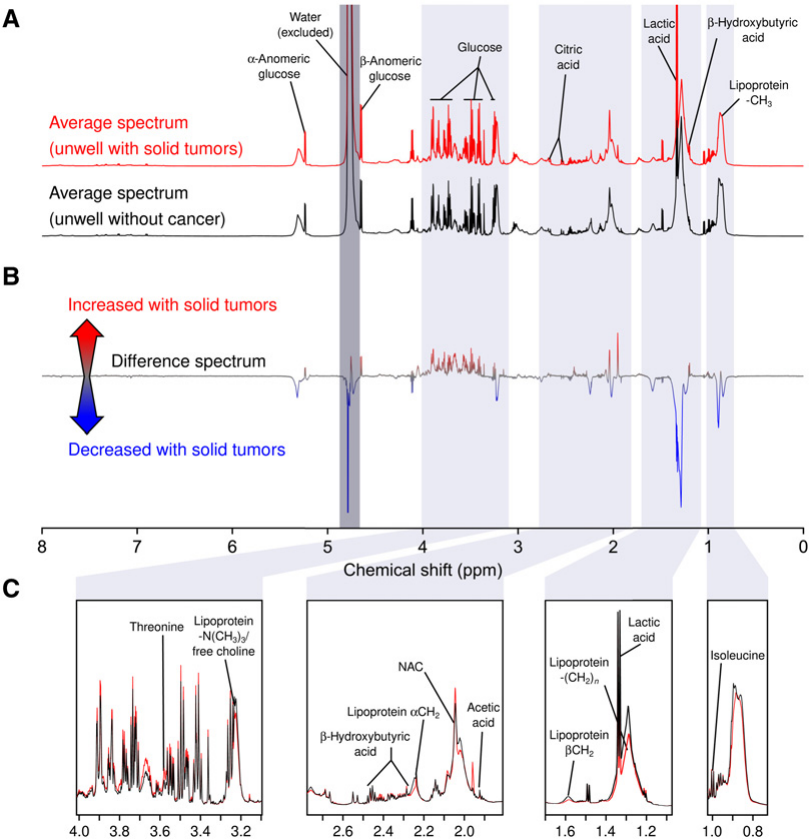
**A,** Mean NMR spectra for samples from unwell patients with either confirmed solid tumor diagnoses (red, *n* = 17) or confirmed noncancer diagnoses (black, *n* = 175). **B,** Difference spectrum showing regions that were increased in patients with solid tumors (red), decreased in patients with solid tumors (blue), or unchanged (gray). **C,** Insets showing magnified regions at points of significant difference between unwell with solid tumor spectra (red) and unwell without cancer spectra (black). NAC, *N*-acetylated glycoproteins.

**Figure 3.**
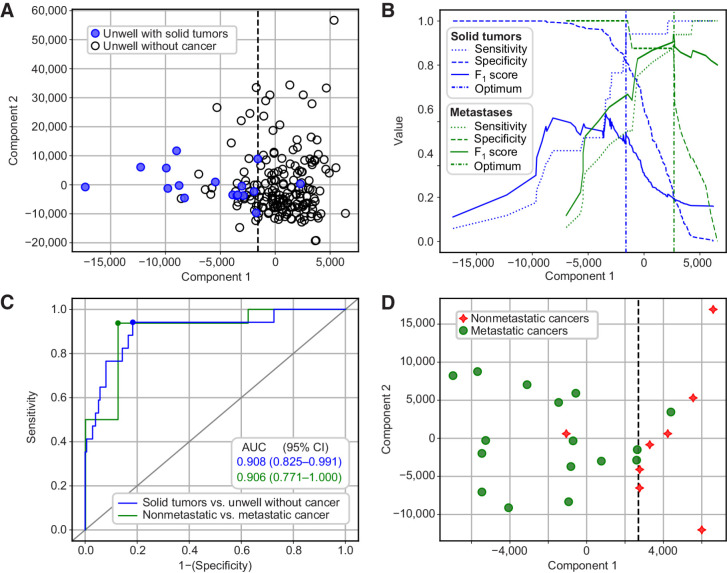
**A,** OPLS-DA plot showing separation of unwell patients with solid tumor diagnoses (blue, filled) from unwell patients with noncancer diagnoses (black, open). **B,** Sensitivity (dots), specificity (dashes), and F_1_ score (continuous) for solid tumors versus unwell patients without cancer (blue), or metastatic versus nonmetastatic cancers (green) at all possible thresholds of classification according to Component 1. Vertical dashed lines show optimal classification threshold for each model. **C,** ROC curves for classification between unwell with solid tumors versus unwell without cancer diagnoses (blue line; model in **A**), and metastatic versus nonmetastatic diagnoses (green line; model in **C**). Small, colored circles on lines indicate points closest to top-left corner, corresponding to dashed vertical lines in **B**. **D,** OPLS-DA plot showing separation of patients with nonmetastatic cancer diagnoses (red stars) or metastatic cancer diagnoses (green circles).

Mean NMR spectra from unwell patients with nonspecific symptoms receiving solid tumor and noncancer diagnoses are shown in Fig. 2, along with the difference spectrum between the two groups. OPLS-DA was able to separate unwell patients with solid tumor diagnoses from unwell patients with noncancer diagnoses (Fig. 3A and B) using the plasma metabolome with an AUC of 0.91 (95% CI: 0.83–0.99; *P* < 0.001; cut-off value Fig. 3B; ROC curve Fig. 3C). Cross-validation and permutation testing confirmed that the model significantly out-performed random chance (*P* < 0.001; Supplementary Fig. S2) confirming that the model performance is not a result of overfitting and that the developed model should be robust to novel data. Indeed, the model was able to identify cancer in the modeling set with sensitivity of 94% (95% CI: 73–99), a specificity of 82% (95% CI: 75–87), a negative predictive value of 99% (95% CI: 96–100), and a balanced accuracy of 88% (95% CI: 85–91). Two by two contingency tables are given in Supplementary Table S2 and complete summaries of model quality metrics at different classification thresholds are presented in Supplementary Fig. S3.

**Figure 4.**
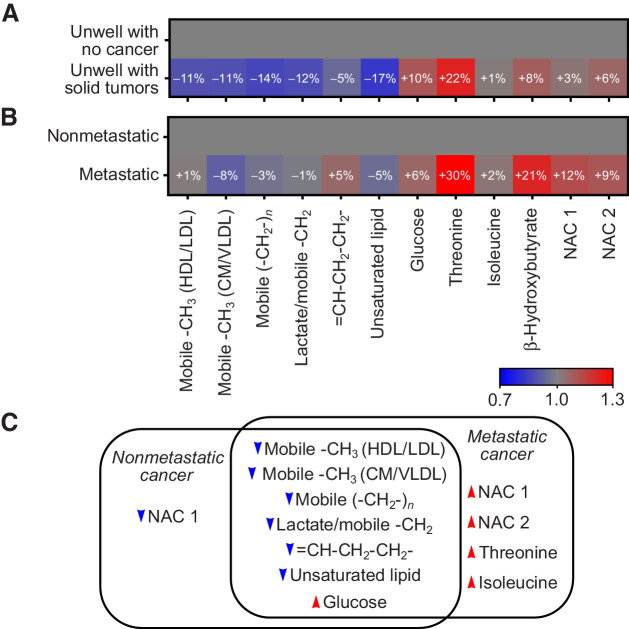
**A,** Fold changes in key metabolites identified by multivariate analysis concentrations in unwell patients with solid tumors relative to the mean metabolite concentrations in unwell patients without cancer. **B,** Fold changes in key metabolite concentrations in patients with metastatic cancer, relative to the mean metabolite concentration in patients with nonmetastatic cancer. **C,** Venn diagram illustrating direction of metabolite concentration changes in metastatic and nonmetastatic cancers, relative to unwell patients without cancer. HDL, high-density lipoprotein. Note that “/” represents that the two metabolites overlap in the NMR data, and not a ratio of the two metabolite concentrations. Individual plots of metabolite concentrations are given in Supplementary Fig. S4.

Inspection of model VIPs revealed that patients with solid tumors had significantly decreased plasma lipoprotein levels [–CH_3_, (–CH_2_–)*_n_*, =CH–CH_2_–CH_2_–, and saturated lipid resonances] along with an increase in plasma glucose, *N*-acetylglucosamine (NAC1), and threonine concentrations compared with unwell patients without cancer in the nonspecific symptom cohort (Fig. 4A; individual metabolite graphs with univariate analyses are shown in Supplementary Fig. S4). Correlation analyses, in the unwell with no cancer cohort, between age and each identified metabolite found no correlation with more than 5% of variation explained by age. Maximum R^2^ for any correlation was 0.05 for the mobile −CH_3_ chylomicrons/very low-density lipoprotein (CM/VLDL) resonance (*r* = −0.23).

### Robustness of validated metabolomics models to independent test data

Following identification of the optimal diagnostic cutoff for the validated “solid tumor versus noncancer” model, classification performance was investigated on the independent test set.

Overall area under the ROC curve for the independent samples was 0.83 (95% CI: 0.72–0.95), which is not significantly different to that of the modeling set (*P* > 0.05). Although the decreased number of patients in the independent set can increase error, this area under the ROC translated to a sensitivity of 71% (five out of seven cancers detected), a specificity of 70% (60 out of 85 noncancer patients identified as such), and a negative predictive value of 97% (60 noncancer patients of 62 predicted noncancer). These classification results were significantly better than chance (Fisher exact test, *P* < 0.05; Supplementary Table S2).

### Plasma metabolite profile of patients with metastatic cancers are distinct from patients with nonmetastatic cancer

Within the population of 24 patients who had solid tumors, OPLS-DA models were also able to separate patients with a metastatic cancer from those with nonmetastatic cancer with an AUC of 0.91 (95% CI: 0.77–1.00; Fig. 3C and D). This model also had sensitivity, specificity, and overall accuracy significantly better than random chance (*P* < 0.05; Supplementary Fig. S5). Model sensitivity was 94% (95% CI: 72–99), specificity was 88% (95% CI: 53–98), and balanced accuracy was 91% (95% CI: 83–98). Two by two contingency tables are given in Supplementary Table S2 and complete summaries of model quality metrics at different classification thresholds are presented in Supplementary Fig. S6.

Inspection of the VIPs highlighted as significant by the multivariate model indicated that patients with metastatic cancer had elevated levels of threonine, β-hydroxybutyrate, NAC1, and *N*-acetylneuraminic acid (NAC2) resonances along with modest decreases in lipoprotein resonances (–CH_3_ CM/VLDL and unsaturated lipid) relative to patients with nonmetastatic cancers (changes and univariate statistics shown in Fig. 4B). A summary of the directions of change of key metabolites in the metastatic and nonmetastatic cancer cohorts relative to the unwell noncancer patient cohort is given in Fig. 4C.

### Identification of cancer before conventional imaging; two case studies

**Figure 5.**
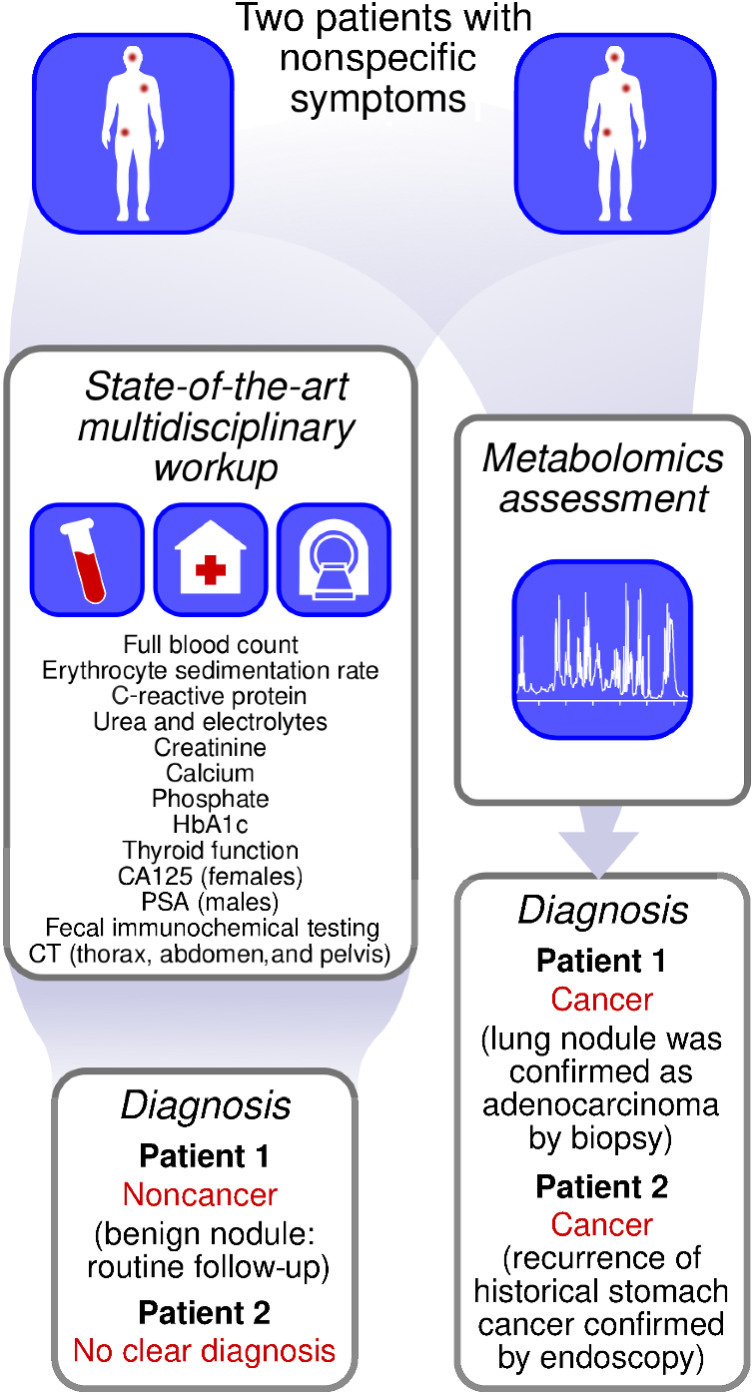
Example patient journeys showing how metabolomics and multidisciplinary diagnostic center workup provided different diagnoses.

Next, we investigated whether the metabolomics model was able to identify early-stage cancers before conventional imaging. The patient cohort was followed up for 1 year to determine whether any new cancer diagnoses were made in patients who had received noncancer diagnoses in the initial 62-day diagnostic window. From the 267 initial noncancer diagnoses, 5 patients developed solid tumors and 2 developed hematological malignancies (identified at a mean of 7 ± 2 months after initial assessment). Of the 5 patients who developed solid tumors, 2 patients were identified as having cancer by metabolomics assessment of baseline blood samples (taken at the point of CT scan and MDC assessment; Fig. 5). Full prediction scatter plots are given in Supplementary Fig. S7.

In the first case, a patient in their 60s was referred to the SCAN pathway because of abdominal pain, anemia, and a raised C-reactive protein level. The radiology identified a nonspecific lung nodule, but metabolomics predicted cancer at baseline. The patient entered routine CT surveillance for the lung nodule. Suspicious features on the surveillance scans prompted a tissue biopsy which confirmed adenocarcinoma. The patient subsequently had a lobectomy 9 months after the initial MDC assessment.

In the second case, a patient in their 80s was referred to the SCAN pathway because of unexplained weight loss. The patient had a past medical history of gastric cancer, treated with distal gastrectomy 11 years prior to referral to SCAN. There was no evidence of recurrence on either the CT imaging or any of the other diagnostic tests conducted as part of the MDC assessment and the patient received a noncancer diagnosis. Recurrence of the gastric cancer was subsequently identified by endoscopy 7 months after initial referral, and it was discovered that the baseline metabolomics data for this patient also clearly predicted cancer.

## Discussion

In this study we have shown that metabolomic analysis of blood can sensitively and specifically identify solid tumors in patients with nonspecific symptoms. We have demonstrated that our models perform consistently under an extensive cross-validation scheme and can predict independent sample classifications, despite heterogeneity in cancer types represented. We have further shown that we can distinguish between nonmetastatic and metastatic cancers, within the solid tumor patient population. Finally, we present two cases in which biofluid metabolomics identified the presence of cancer earlier than the initial CT scan.

### Metabolomics detects cancer in patients with nonspecific symptoms

The diagnosis of malignancy in patients presenting with nonspecific symptoms is a problem that is not solved by established population-level screening programs, such as those used for breast cancer, or by specific symptom referral pathways, such as those used to refer a patient with a persistent cough. In many centers there remains no clinical referral pathway for patients with nonspecific symptoms.

Although multidisciplinary approaches, such as that being used in Oxfordshire, have shown promise for cancer diagnosis in patients with nonspecific symptoms (26) they are not established in many centers in the UK or around the world. Without such a pathway, most of the patients with cancer in the cohort studied would probably have remained undetected until they presented at a later stage. Metabolomics analysis of blood is both rapid and inexpensive, and may enable accurate, timely, and cost-effective triaging of patients with suspected cancer.

We have also shown that we can distinguish between metastatic and nonmetastatic cancers, within the solid tumor patient population. This ability to diagnose metastasis is independent of our ability to be able to diagnose the primary tumor and shows promise for the use of metabolomics as a potential cancer staging tool. The ability to identify metastatic patients at first presentation may change the investigations performed and the pathway for these patients.

### Metabolomics has the potential to identify cancers before they are detectable by CT

In our study cohort, 5 patients developed solid tumors within 1 year of receiving a noncancer diagnosis. In 2 of these 5, our baseline metabolomics analysis indicated the presence of cancer before conventional imaging and investigation enabled a diagnosis to be made. This suggests that metabolomics analysis has the potential to detect cancer earlier than the current conventional investigation pathways, and may be of value for both new presentations of malignancy and recurrence. Although the metabolomics did not predict cancer in all 5 of the patients who later went on to a cancer diagnosis, this may reflect one of many factors, including insufficient training data for specific tumor types or tumor size at baseline assessment. Our modeling set was heterogeneous, including a broad range of cancers, but at the expense of depth for individual cancer types. Future studies, with increased numbers of patients in specific cancer subsets, are likely to be needed to accurately determine the sensitivity of our models for early cancer diagnoses.

The case of the patient with the new primary lung tumor offers an interesting insight into how metabolomics may be deployed clinically. This patient's CT demonstrated a small lung nodule, which is a relatively common scenario as these nodules are commonly identified as an incidental abnormality on CT scans performed for a variety of clinical indications. Fortunately, most of these lung nodules are benign, but 2% to 3% will develop into lung cancers (27). Identifying which nodules will develop into cancers in a timely fashion is important for improving outcomes. The British Thoracic Society Guidelines advise serial CT scans for solid lung nodules >5 mm at either 3-, 12-, or potentially 24-monthly intervals over a period of 1 to 2 years (27). By observing changes in the characteristics and size of the nodule, those nodules that are likely to be malignant can be detected. Diagnosis is confirmed with further imaging, such as PET-CT scanning, and potentially a CT-guided lung biopsy, which has a small but significant morbidity and is rightly reserved for those where there is a significant suspicion of malignancy. Interval scanning places an increased burden on radiology departments, and an additional radiation dose and multiple hospital visits for the patient. In this study, we had metabolomic evidence that a small lung nodule of indeterminate etiology at presentation was malignant. This required repeat CT scans to identify nodule enlargement suggestive of malignancy which was then confirmed on biopsy.

### Comparison of metabolomics with alternative technologies

The mainstay of modern clinical cancer diagnosis is imaging, primarily CT scanning, and if positive this is frequently coupled with biopsy. However, it is clear from observing the numbers of late-stage cancer diagnoses (3) that not all patients with cancer are being referred for imaging investigations soon enough. Here we have demonstrated that metabolomics is one technology that could identify these patients sooner, but it is not the only technology in development. One notable alternative technology is the analysis of circulating tumor DNA (ctDNA), which relies on the sequencing of DNA shed from tumors into the blood stream (reviewed in ref. 28). The abundance of particular tumor-associated mutations in the isolated DNA gives an indication of tumor burden. Owing to its genetic approach, ctDNA analysis has great potential for distinguishing tumor subtypes (29) and for monitoring tumor evolution (30). However, for initial detection of tumors the case is less clear, as ctDNA is released only by tumor cells. Consequently, there is no indirect amplification of the signal as is seen with metabolomics. Instead ctDNA analysis must detect the minute traces of DNA released directly by tumor cells which has intrinsic sensitivity limitations. A second factor is that all mutations must be known *a priori*, meaning that some mutations may be missed, even if they are being released by tumor cells.

### Theoretical mechanism for cancer-mediated metabolic disruption

Dysregulated metabolism is so inextricably linked with cancer that it is included as one of the key hallmarks of cancer (31), and the study of oncometabolites and altered metabolism are major areas of research (32, 33). As such, it may be logical to assume that the metabolic changes we observe in this study are direct consequences of intracellular changes in cancer cells, for example there is a growing body of evidence that lipid metabolism is disrupted directly in tumor cells (reviewed in refs. 34, 35), which also has a direct impact on intracellular glucose concentrations. However, the small size of some of the tumors would limit their direct effect on the overall whole-body metabolic profile. Therefore, we expect that the metabolic profile changes we observe are the combined sum of changes associated with tumor metabolism and altered stromal metabolism—the so-called reactive stroma (36, 37). Others have shown that stromal signaling pathways can be activated by cancerous epithelial cells to promote the transition to a cancer-associated reactive stroma, which is thought to generate a supporting microenvironment for tumor growth and progression (38). Unfortunately, in most investigations metabolites have been studied in samples from individuals with specific types of cancer and then compared with those in healthy controls, which makes direct comparison between our findings and those of others problematic. However, some generalizations are possible and largely agree with our observations.

In this study, we see clear trends of change underpinning separations. Given that patients were fasted for at least 2 hours prior to sample collection and that there's no link to BMI, it's likely that these changes are metabolic responses. One clear trend is for decreased lipid concentrations being associated with solid tumors. Although intracellular changes in lipid metabolism do occur, we believe that the systemic response to the presence of the tumor is a more likely source of the altered lipid metabolism (39). For example, it is often argued that lipoproteins are an important source of metabolites for tumors and this demand may account, at least in part, for the lipid changes we have observed (39, 40). Another example is the association of low levels of low-density lipoprotein (LDL) with poor outcomes in patients with liver cancer (41) and lower levels of VLDL with breast carcinoma compared with healthy controls (42). Inflammatory tumor-associated cytokines like TNF, IL1β, IL6, and monocyte chemoattractant protein-1 (MCP-1) are all known to be involved in altered lipid metabolism at a systemic level (43). In particular, IL6 has been shown to reduce insulin-induced lipogenesis (44), whilst IL1β stimulated lipid accumulation by the liver (45). This involvement of cytokines lends credence to the hypothesis that it is indirect systemic changes, both within the local tumor microenvironment and across the whole body, e.g., changes in liver metabolism, that may be driving the magnification of the cancer metabolome signal. This indirect magnification hypothesis agrees with previous mouse studies, in which very small tumors growing in different locations in the body produced markedly different metabolic profile changes that cannot be simply attributed to any ‘direct’ metabolic effects they could have produced (6). These systemic effects have been postulated for some time (46), but it is only relatively recently that suitable whole-organism studies in cancer have been carried out to explicitly investigate the link between tumor and systemic metabolism (47, 48).

The increase in glucose concentrations in cancer and the increase in threonine in metastatic disease are more counterintuitive based on the Warburg effect. However, we are looking at systemic, not intratumoral glucose concentrations, and increased circulating glucose concentrations have often been associated with an increased risk of developing cancer (49, 50). Moreover, changes in plasma glucose concentration may reflect release from muscle glycogen stores in response to situations of stress (48).

Overall, we believe that the profile of altered metabolism we observe is a combination of multiple interactions involving the tumor, the local microenvironment, and distant regions such as the liver and adipose tissue, responding to the altered inflammatory profile induced by the tumor.

### Clinical application of metabolomics

For metabolomics to make the transition from a research project to a clinically useful tool, it needs to: (i) have sufficient sensitivity and specificity to detect tumors in a cohort of patients; (ii) enable alterations in investigation pathways for patients with suspected malignancies, including the cohort investigated here of “low-risk, but not no-risk” patients.

Incorporating metabolomics into the initial investigation of patients with nonspecific symptoms may assist in the triage of patients for further referral or investigation. If metabolomic analysis is ordered from primary care with other initial blood analyses, it would provide valuable information for next diagnostics steps. Practically speaking a metabolomics blood test would require a blood sample collection and then analysis at a specialist center, meaning results could be returned before imaging appointments. Overall cost would be considerably lower than imaging-based investigations, with a likely price around that of other moderately specialist blood investigations.

At the optimum classification threshold, our negative predictive value of 99% provides a valuable “rule-out” test for cancer that would be useful for primary care physicians, letting them reassure their patients. Although specificity in our model is high, the low percentage of true cancers in the at-risk population would lead to false positives. A positive test, therefore, is a strong indication for referral for imaging investigation, but not a guarantee of cancer. In such cases, it will be necessary for the physician to take into consideration both the metabolomic prediction of cancer and other symptoms. In a larger study, it may be possible to formalize this framework into a risk score to help diagnoses. In any case, even a strong suspicion of cancer often justifies the nonnegligible radiation dose from a CT scan. In our study, for every patient with cancer that we would recommend for CT scanning, we would also recommend CT scans for 2 patients without cancer. However, we would also eliminate 9 patients from cancer imaging pathways.

In a more general sense, metabolomic analysis may help interpret the significance of incidental findings on imaging studies, such as lung nodules or pancreatic cysts, prompting earlier interventions than the current watch-and-wait approach. This may enable an earlier diagnosis to be reached, reducing the need for surveillance imaging, radiation exposure, and costs. The ability of metabolomics to differentiate metastatic from nonmetastatic cancer may be clinically important, providing a guide on the most appropriate investigations, improving diagnostic pathway efficiency, with potential significant cost and time savings.

### Conclusions and future directions

In this study we have shown that the application of NMR metabolomics to a small blood sample offers a complementary approach to the current pathway for investigating patients with a clinical suspected possible malignancy. It is sensitive, specific, and of low cost, requiring nothing more than a blood sample in the clinic and an inexpensive NMR analysis, and can identify patients with solid tumors when referred with nonspecific symptoms—a traditionally hard-to-diagnose cohort.

NMR metabolomics now needs testing in a larger cohort of patients, potentially linked to multiple specific cancer referral pathways, as well as the pathway for the “low-risk, but not no-risk” patient group. By including potential time and cost-savings in the analysis, as well as the conventional cancer detection utility metrics, the utility of NMR metabolomics can be evaluated in a broader fashion.

## Supplementary Material

Supplementary Data
