# Supplementary material for: Metabolomic Biomarkers in Blood Samples Identify Cancers in a Mixed Population of Patients with Nonspecific Symptoms
Source: Clin Cancer Res. 2022 Jan 4;28(8):1651–61. doi: 10.1158/1078-0432.CCR-21-2855 (PMC7613224; doi:10.1158/1078-0432.CCR-21-2855)
Supplement: Supplementary Data [file ccr-21-2855_supp1.pdf]

Supplementary information

To accompany "Metabolomics identifies cancers in a mixed population of patients with non-specific symptoms", by James R. Larkin, Susan Anthony, Vanessa A. Johanssen, Tianrong Yeo, Megan Sealey, Abi G. Yates, Claire Friedemann Smith, Timothy D. W. Claridge, Brian D. Nicholson, Julie-Ann Moreland, Fergus Gleeson, Nicola R. Sibson, Daniel C. Anthony, Fay Probert

Email: fay.probert@chem.ox.ac.uk

Contents

Supplementary NMR methods 2

Supplementary statistical methods 2

Wilson Score Interval calculation 2

Comparison of male:female ratios 2

Comparison of ROC curves 2

Table SI1: List of all cancers present in the patient population 4

Table SI2: 2x2 contingency tables 4

Figure SI1: Distribution of patient BMI across the unwell with solid tumours vs unwell without cancer model 5

Figure SI2: Unwell with solid tumours vs. unwell without cancer model validation plots 6

Figure SI3: Unwell with solid tumours vs. unwell without cancer model quality metrics 7

Figure SI4: Univariate statistical plots for key metabolites 8

Figure SI5: Metastatic cancer vs. non-metastatic cancer model validation plots 9

Figure SI6: Metastatic cancer vs. non-metastatic cancer model quality metrics 10

Figure SI7: Prediction of patients developing cancer within one year 11

Supplementary NMR methods

Full NMR data pre-processing methods

While all spectra were visually inspected in Topspin for errors in baseline correction, referencing, spectral distortion or contamination, only CPMG spectra were used for statistical analysis. Acquired free induction decays (FIDs) were zero-filled by a factor of 2 and multiplied by an exponential function corresponding to 0.3Hz line broadening prior to Fourier transformation. All spectra were phased, baseline corrected (using a 3rd degree polynomial) and referenced to the lactate-CH_3 doublet resonance at δ = 1.33ppm using Topspin 4.0 (Bruker, Germany; RRID:SCR_014227) and then imported into Matlab (Mathworks, MA, USA; RRID:SCR_001622). The region between 0.29-9.68ppm, excluding the region covering the residual water peak from 4.67 to 4.89ppm, was divided into 0.01ppm width 'buckets' and integrated.

Supplementary statistical methods

Wilson Score Interval calculation

Upper and lower confidence intervals for proportions from a contingency table were calculated using the Wilson Score Interval:

$$\frac{p+\frac{u}{2}}{u+1} \pm \frac{z_{crit}}{u+1} \cdot \sqrt{\frac{p(1-p)}{n}+\frac{u}{4n}}$$

where

$$u = z_{crit}^2/n$$

and p is the proportion from the contingency table, z_{crit} at α=0.05 is 1.96 for a 2-sided normal distribution, and n is the sample size.

Comparison of male:female ratios

Comparisons of male:female sex ratios were carried out by calculation of the *Z*-statistic for each ratio and comparing to the expected normal distribution. First, Z for each ratio was calculated using:

$$Z = \frac{(p_1 - p_2)}{\sqrt{p(1-p)(\frac{1}{n_1}+\frac{1}{n_2})}}$$

where p_1 is the proportion of male:female in group 1, p_2 is the proportion of male:female in group 2, p is the overall proportion of male:female in both groups combined, n_1 is the number in group 1, and n_2 is the number in group 2.

The absolute *Z*-statistic was then compared to a normal distribution with a mean of 0 and a standard deviation of 1, and a two-sided *p*-value was calculated.

Comparison of ROC curves

To compare two different ROC curves, a *Z*-statistic was calculated based upon the areas under each curve and the standard error of each area using:

$$Z = \frac{|A_1 - A_2|}{\sqrt{SE_{A1}^2 + SE_{A2}^2}}$$

where A_1 and A_2 are the areas under ROC curves 1 and 2 respectively, and SE_{A1} and SE_{A2} are the standard errors of the areas under ROC curves 1 and 2 respectively. The absolute *Z*-statistic was then compared to a normal distribution with a mean of 0 and a standard deviation of 1, and a two-sided *p*-value was calculated.

Table SI1: List of all cancers present in the patient population

Type of cancer	Number (of which in modelling; independent test sets)
Large bowel	8 (4;4)
Lung	5 (3;2)
Pancreas	3 (3;0)
Breast	2 (1;1)
Bladder	1 (1;0)
Gall bladder	1 (1;0)
Gastrointestinal stromal tumour	1 (1;0)
Kidney	1 (1;0)
Ovary	1 (1;0)
Pancreas/stomach	1 (1;0)
TOTAL	24 (17;7)

Table SI2: 2x2 contingency tables

Table SI2A: Solid tumour *vs.* non-cancer model

Predicted class	True class	
	Solid tumour	Non-cancer
Solid tumour	16	32
Non-cancer	1	143

Table SI2B: Metastatic *vs.* non-metastatic cancer model

Predicted class	True class	
	Metastatic cancer	Non-metastatic cancer
Metastatic cancer	15	1
Non-metastatic cancer	1	7

Table SI2C: Independent test set predictions for solid tumour *vs.* non-cancer model

Predicted class	True class	
	Solid tumour	Non-cancer
Solid tumour	5	25
Non-cancer	2	60

Figure SI1: Distribution of patient BMI across the unwell with solid tumours vs unwell without cancer model

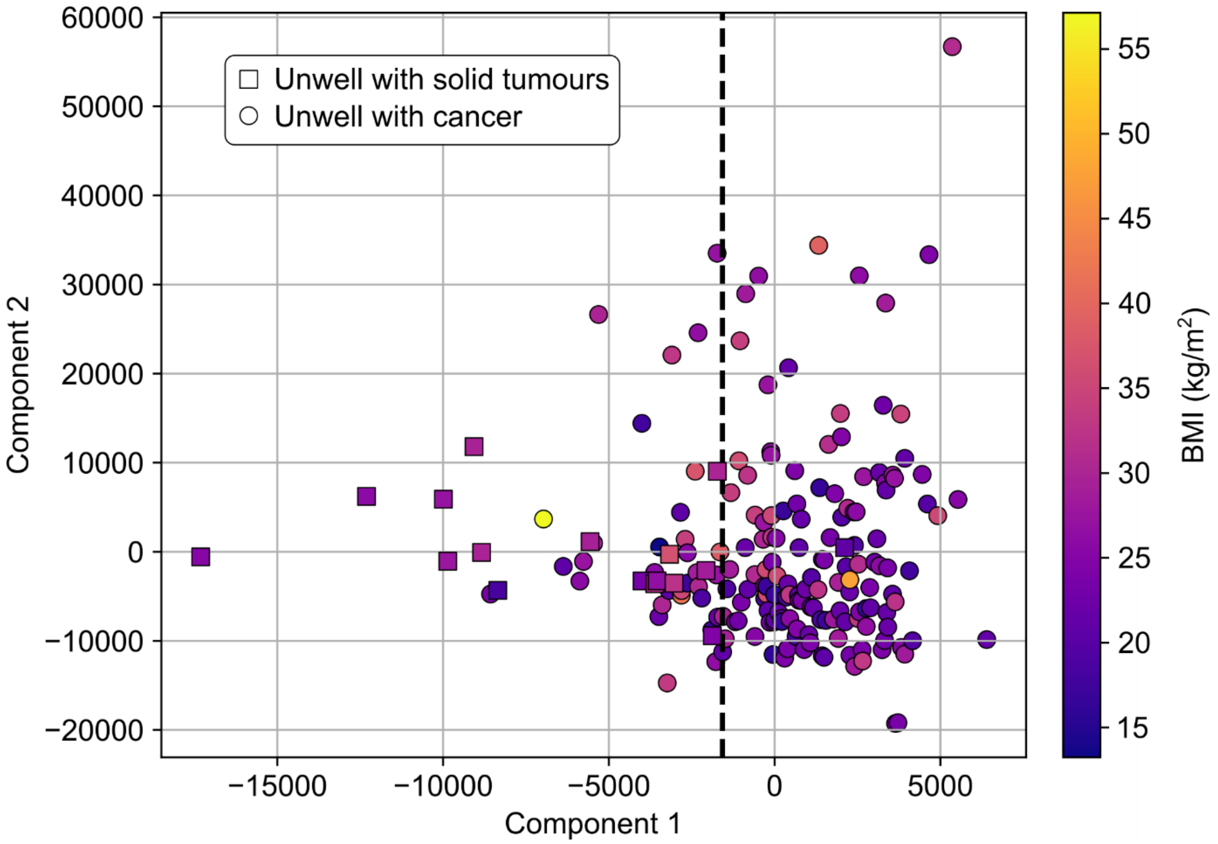

OPLS-DA plot showing separation of unwell patients with solid tumour diagnoses (squares) from unwell patients with non-cancer diagnoses (circles), coloured according to BMI for each patient where available (n=187 out of 192 patients in this model). R^2 for correlation between BMI and Component 1 is 0.025 (Pearson's).

Figure SI2: Unwell with solid tumours vs. unwell without cancer model validation plots

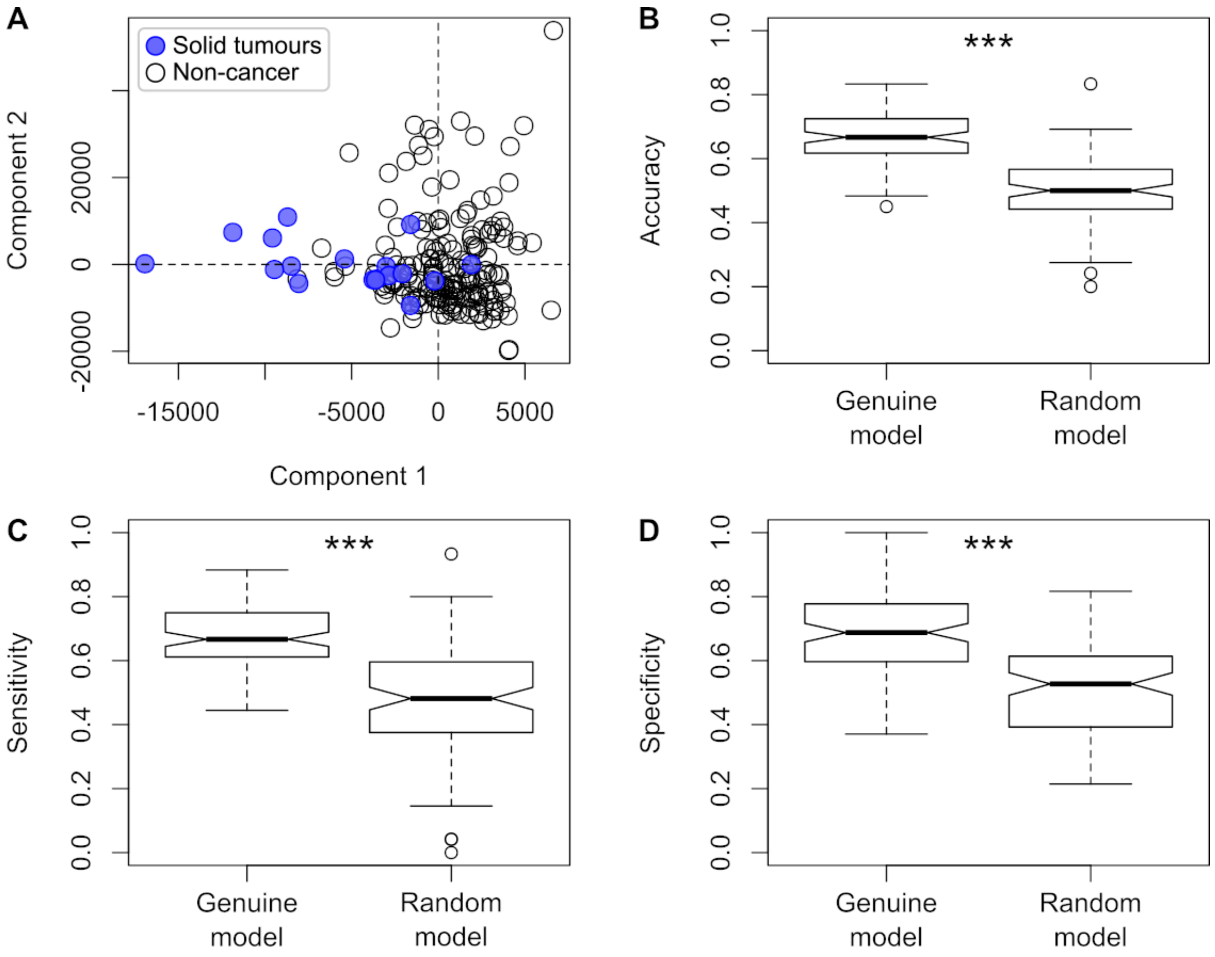

A: OPLS-DA plot showing separation of unwell patients with solid tumours diagnoses (blue) from unwell patients with non-cancer diagnoses (open). B-D: Accuracy, sensitivity and specificity for models generated using both subsets of patients with correct group assignments (genuine models) and subsets of patients with random group assignments (random models). *** p<0.001, Kolmogorov–Smirnov test.

Figure SI3: Unwell with solid tumours vs. unwell without cancer model quality metrics

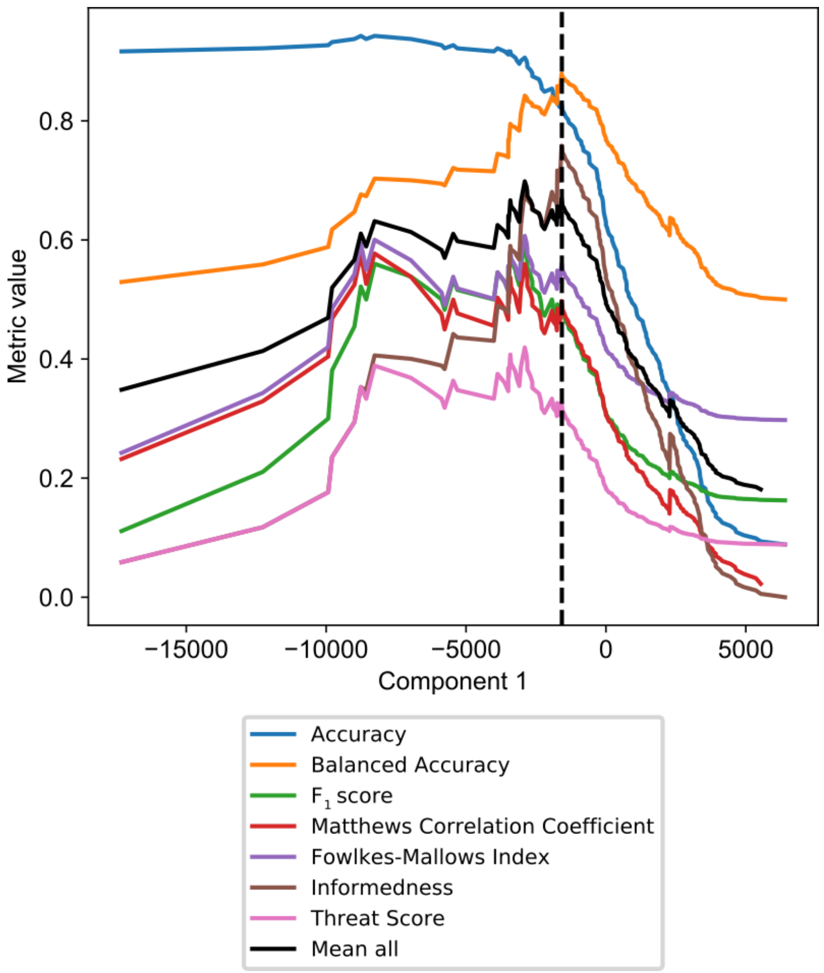

Comparison of seven model classification metrics at different thresholds of Component 1 for the unwell with solid tumours *vs.* unwell without cancer model. Mean of all the metrics is shown in the bold black line. Vertical dashed line represents the chosen optimal model classification threshold.

Accuracy: $Accuracy = \frac{\text{TP+ TN}}{\Sigma \text{ Total population}}$

Balanced accuracy: $BA = \frac{\text{Sensitivity+Specificity}}{2}$

F_1 score: $F_1 = \frac{\text{2TP}}{\text{2TP+FP+FN}}$

Matthews correlation coefficient: $MCC = \frac{\text{TP} \times \text{TN}-\text{FP} \times \text{FN}}{\sqrt{(\text{TP+FP})(\text{TP+FN})(\text{TN+FP})(\text{TN+FN})}}$

Fowlkes-Mallows index: $FM = \sqrt{\frac{TP}{TP+FP} \cdot \frac{TP}{TP+FN}}$

Informedness: $I = Sensitivity + Specificity - 1$

Threat score: $TS = \frac{TP}{TP+FN+FP}$

Figure SI4: Univariate statistical plots for key metabolites

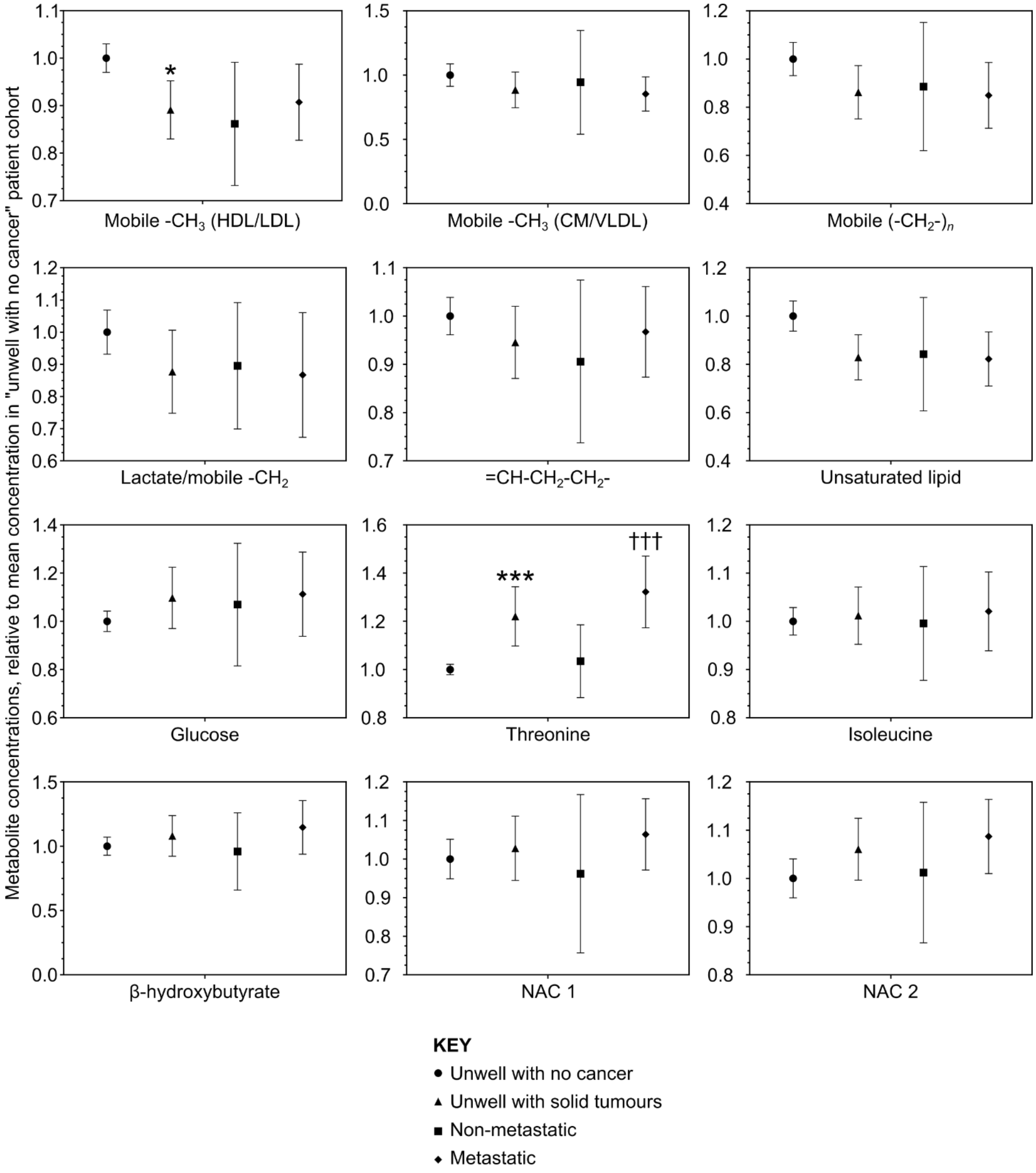

Plots showing mean ± 95% confidence intervals for metabolites listed in Figure 4. Univariate analysis statistics: *=p<0.05, ***=p<0.001, t-test between the solid tumour and non-cancer groups. †††=p<0.001, t-test between non-metastatic and metastatic cancer groups.

Figure SI5: Metastatic cancer vs. non-metastatic cancer model validation plots

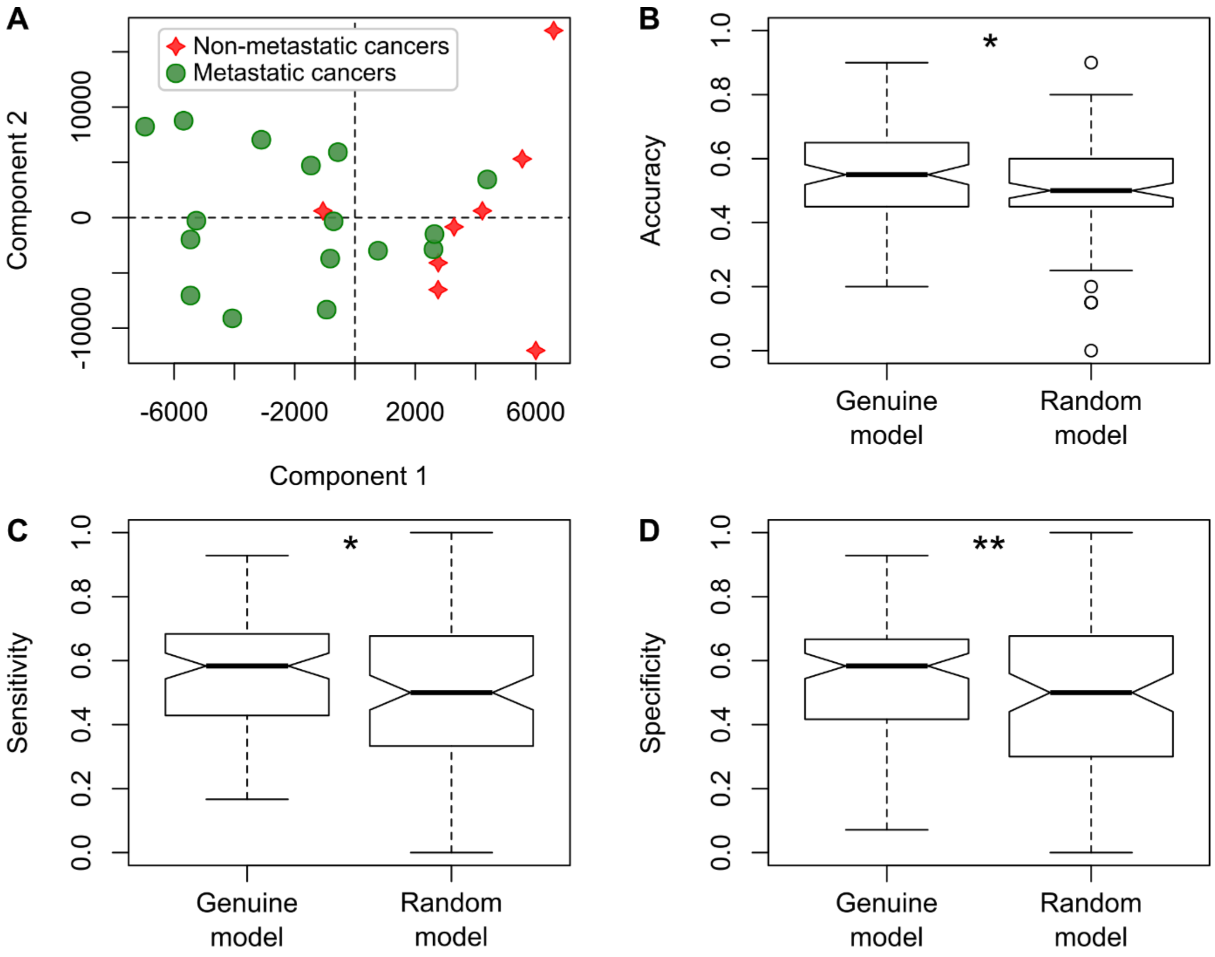

A: OPLS-DA plot showing separation of patients with metastatic cancer diagnoses (green circles) from patients with non-metastatic cancer diagnoses (red stars). B-D: Accuracy, sensitivity and specificity for models generated using both subsets of patients with correct group assignments (genuine models) and subsets of patients with random group assignments (random models). * p<0.05; ** p<0.01, Kolmogorov–Smirnov test.

Figure SI6: Metastatic cancer vs. non-metastatic cancer model quality metrics

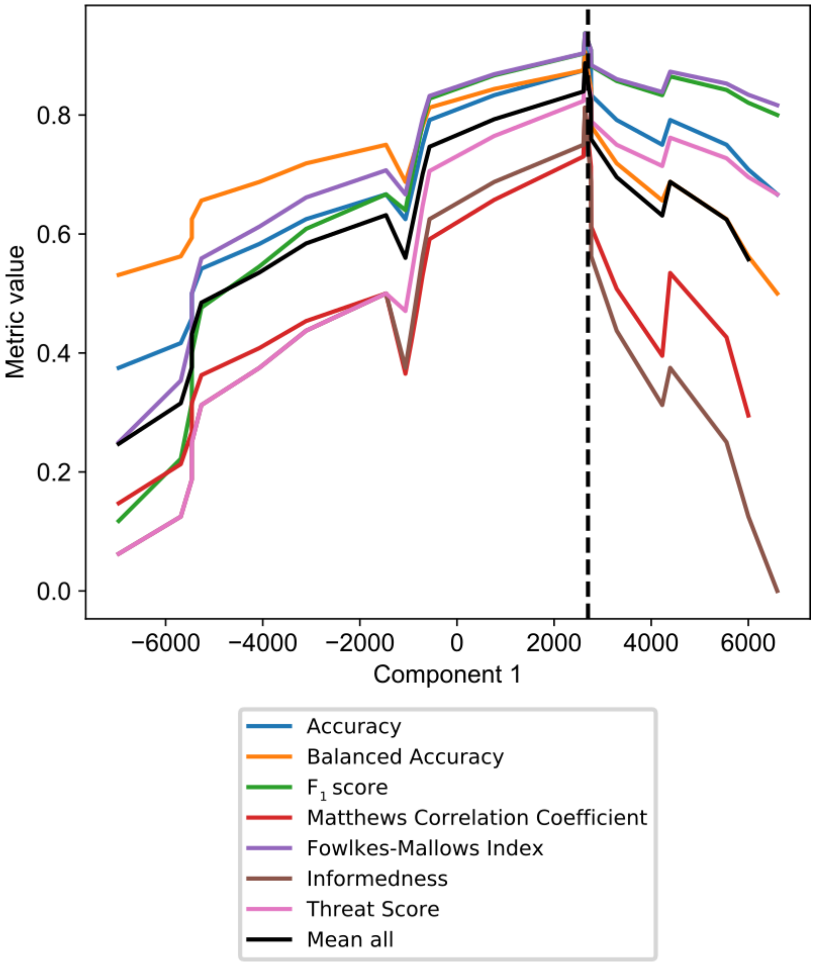

Comparison of seven model classification metrics at different thresholds of Component 1 for the metastatic cancer *vs.* non-metastatic cancer model. Mean of all the metrics is shown in the bold black line. Vertical dashed line represents the chosen optimal model classification threshold.

Accuracy: $Accuracy = \frac{\mathrm{TP+\ TN}}{\Sigma\ \mathrm{Total\ population}}$

Balanced accuracy: $BA = \frac{\mathrm{Sensitivity+Specificity}}{2}$

F_1 score: $F_1 = \frac{\mathrm{2TP}}{\mathrm{2TP+FP+FN}}$

Matthews correlation coefficient: $MCC = \frac{\mathrm{TP \times TN - FP \times FN}}{\sqrt{\mathrm{(TP+FP)(TP+FN)(TN+FP)(TN+FN)}}}$

Fowlkes-Mallows index: $FM = \sqrt{\frac{TP}{TP+FP} \cdot \frac{TP}{TP+FN}}$

Informedness: $I = Sensitivity + Specificity - 1$

Threat score: $TS = \frac{TP}{TP+FN+FP}$

Figure SI7: Prediction of patients developing cancer within one year

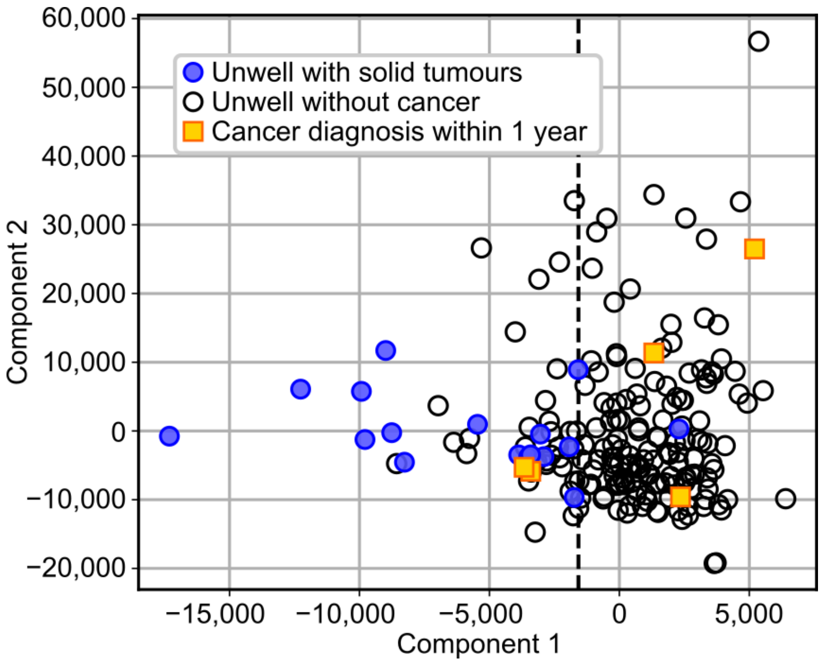

OPLS-DA plot showing prediction of the five patients who developed solid tumours within one year of a non-cancer diagnosis (yellow squares, filled) superimposed upon the separation of unwell patients with solid tumour diagnoses (blue circles, filled) from unwell patients with non-cancer diagnoses (black circles, open). Patients lying left of the dashed vertical line are predicted to have cancer.
